# Epstein–Barr Virus (EBV) Rta-Mediated EBV and Kaposi's Sarcoma-Associated Herpesvirus Lytic Reactivations in 293 Cells

**DOI:** 10.1371/journal.pone.0017809

**Published:** 2011-03-10

**Authors:** Yen-Ju Chen, Wan-Hua Tsai, Yu-Lian Chen, Ying-Chieh Ko, Sheng-Ping Chou, Jen-Yang Chen, Su-Fang Lin

**Affiliations:** 1 College of Medicine, Graduate Institute of Microbiology, National Taiwan University, Taipei, Taiwan; 2 National Institute of Cancer Research, National Health Research Institutes, Miaoli County, Taiwan; Duke University Medical Center, United States of America

## Abstract

Epstein–Barr virus (EBV) Rta belongs to a lytic switch gene family that is evolutionarily conserved in all gamma-herpesviruses. Emerging evidence indicates that cell cycle arrest is a common means by which herpesviral immediate-early protein hijacks the host cell to advance the virus's lytic cycle progression. To examine the role of Rta in cell cycle regulation, we recently established a doxycycline (Dox)-inducible Rta system in 293 cells. In this cell background, inducible Rta modulated the levels of signature G1 arrest proteins, followed by induction of the cellular senescence marker, SA-β-Gal. To delineate the relationship between Rta-induced cell growth arrest and EBV reactivation, recombinant viral genomes were transferred into Rta-inducible 293 cells. Somewhat unexpectedly, we found that Dox-inducible Rta reactivated both EBV and Kaposi's sarcoma-associated herpesvirus (KSHV), to similar efficacy. As a consequence, the Rta-mediated EBV and KSHV lytic replication systems, designated as EREV8 and ERKV, respectively, were homogenous, robust, and concurrent with cell death likely due to permissive lytic replication. In addition, the expression kinetics of EBV lytic genes in Dox-treated EREV8 cells was similar to that of their KSHV counterparts in Dox-induced ERKV cells, suggesting that a common pathway is used to disrupt viral latency in both cell systems. When the time course was compared, cell cycle arrest was achieved between 6 and 48 h, EBV or KSHV reactivation was initiated abruptly at 48 h, and the cellular senescence marker was not detected until 120 h after Dox treatment. These results lead us to hypothesize that in 293 cells, Rta-induced G1 cell cycle arrest could provide (1) an ideal environment for virus reactivation if EBV or KSHV coexists and (2) a preparatory milieu for cell senescence if no viral genome is available. The latter is hypothetical in a transient-lytic situation.

## Introduction

Human oncogenic herpesviruses such as Epstein–Barr virus (EBV) and Kaposi's sarcoma-associated herpesvirus (KSHV) are closely linked to a variety of malignancies including nonkeratinizing nasopharyngeal carcinoma, gastric adenocarcinoma, Burkitt's lymphoma, Kaposi's sarcoma, primary effusion lymphoma, multicentric Castleman's disease, and various forms of lymphoproliferative disorders. Both EBV and KSHV are latent residents in B lymphocytes and show sporadic reactivation in lymphoepithelial tissues such as tonsils [1], [2], [3]. Lytic reactivation of EBV or KSHV in epithelial cells of the nasopharynx is strongly influenced by the state of differentiation [4], [5], [6]. In addition, XBP-1s, a product of the master gene responsible for B cell differentiation, was recently suggested to be one of the physiological stimuli that trigger the lytic switch of EBV and KSHV in latently infected B cells [7], [8].

For a cycling cell, growth arrest in the G1 phase implies one of the following fates to choose: quiescence (re-enters proliferation at a later time), apoptosis, differentiation or senescence [9], [10]. Among these four outcomes, differentiation and senescence share two features in common: dramatic chromosome remodeling [11] and lengthy development time (usually days). Cell senescence is a biochemical process exhibited by metabolically active cells whose cell cycles are frozen beyond the restriction point in G1 phase. First identified in *in vitro* cultured cells, cellular senescence occurs both in primary and cancer cell lines [12], [13]. In addition, the limit in proliferative capacity triggered by aberrant mitogenic signals of oncogenes, known as oncogene-induced senescence (OIS), is an alternative tumor suppressive mechanism that has been recently validated *in vivo* [14], [15]. Senescence not only occurs in pre-malignant cells, but also appears in malignant tumors. In the latter case, senescence was usually produced by the removal of an essential oncogenic stimulus or the restoration of a tumor suppressor. For example, ablation of c-Myc in transgenic mouse models induced rapid tumor regression associated with senescence [16]; systemic expression of a dominant-interfering Myc mutant in a preclinical mouse model with Ras-initiated lung adenocarcinoma triggered rapid tumor regression accompanied by senescence [17].

Lytic replication of herpesvirus occurs preferentially in the G1 phase of the cell cycle [18]. Accumulating evidence indicates that a number of viral immediate-early proteins actively exert a growth-arrest function by which the virus induces a less competitive environment for resources required for viral DNA replication. In this regard, when EBV undergoes lytic replication, host cells are protected from apoptosis and the DNA-synthetic machinery is blocked, although the activities of certain S-phase regulators increase [19], [20]. In addition, upon viral infection, ICP0 of herpes simplex virus induces cell cycle arrest in G1 by both p53-mediated and p53-independent pathways [21]. Through the up-regulation of cyclins E and D, the IE2 protein of human cytomegalovirus potently arrests U373 cells and simultaneously blocks cellular DNA synthesis [22]. Conceivably, G1 phase is not only a pivotal stage for cell fate determination [9], [10], but also is critical for virus fate, namely to maintain latency or to initiate a lytic replication episode.

Among the identified immediate-early molecules, RTA (replication and transcription activator) is positionally and structurally conserved among the genomes of all gamma-herpesviruses. Ectopic expression of the EBV Rta in epithelial or B cells is capable of efficiently inducing the lytic cycle of EBV [23], [24]. Similarly, ectopic expression of KSHV RTA (K-RTA) in B or endothelial cells latently infected with KSHV leads to the successive expression of KSHV early and late genes [25], [26]. We recently established doxycycline-inducible system of Rta in 293 cells, nasopharyngeal carcinoma cells (NPC-TW01), and laryngeal carcinoma cells (HEp-2). We found that, in the absence of BZLF1 and other EBV viral proteins, Rta alone can promote irreversible G1 arrest followed by cellular senescence in these epithelial cells ([27] and unpublished). In the present study, we further demonstrate that in 293 cells, the doxycycline-inducible Rta not only reactivates EBV but also KSHV, to similar efficacy. While the precise mechanism of Rta-mediated KSHV reactivation is currently not resolved, results from comparative kinetics studies strongly indicate a casual role of Rta-induced G1 arrest in EBV and KSHV reactivations.

## Results

### EBV Rta alone is sufficient to initiate and complete lytic EBV replication in EREV8 cells

**Figure 1:**
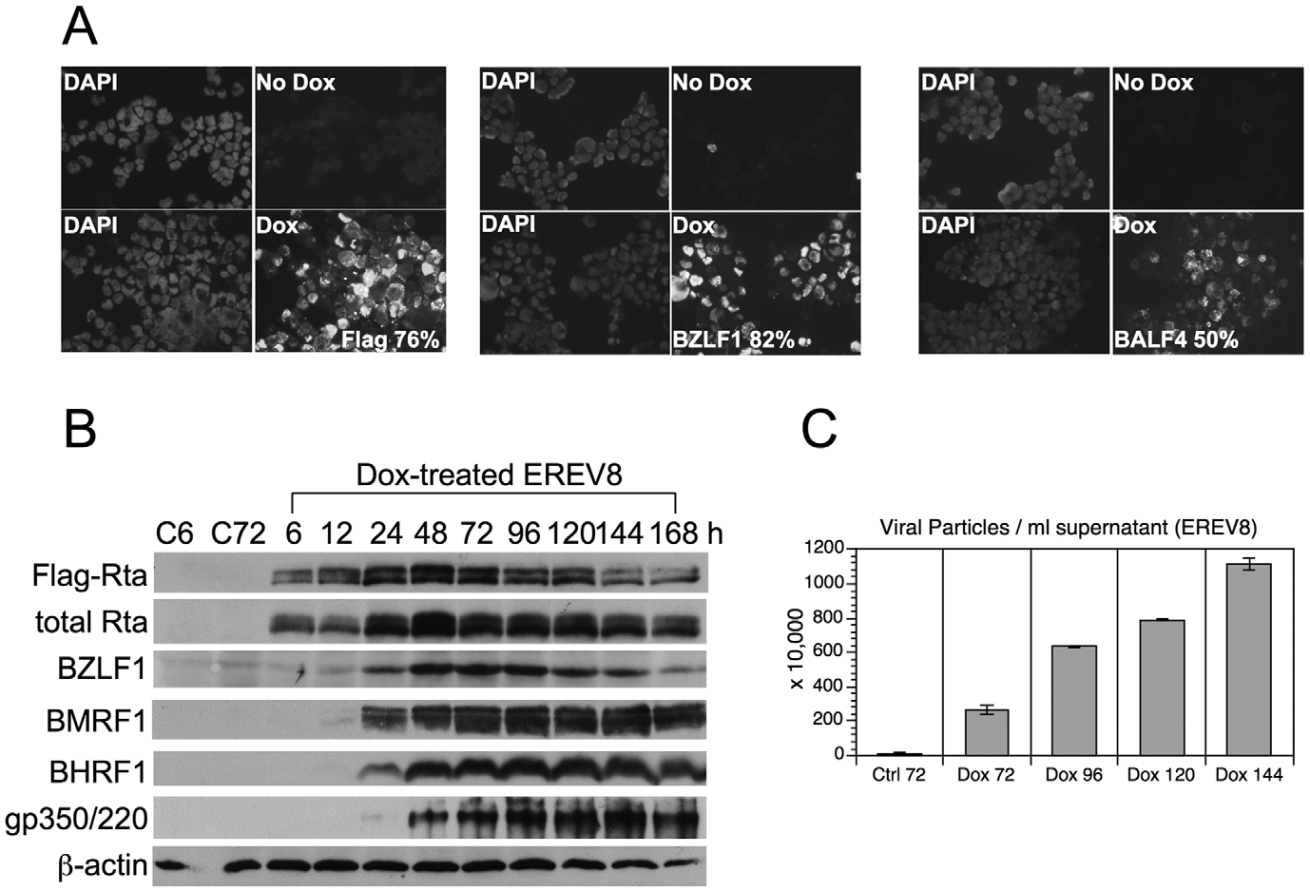
Reactivation of EBV lytic replication by EBV Rta in EREV8 cells. (A) Individual induction efficiency of Flag-EBV Rta (Flag), BZLF1, and late glycoprotein BALF4/gB were shown by an immunofluorescence assay. Untreated (–) or EREV8 cells treated with doxycycline (Dox) for 48 h were analyzed in parallel. Cells with immuno-positivity were quantified by flow cytometry and indicated in percentages for each detection. (B) Expression kinetics of EBV lytic proteins including BZLF1, BMRF1, BHRF1, and gp350/220 in Dox-induced EREV8 cells for the indicated times were analyzed by western blot analysis. β-actin served as a loading control. C6 and C72 indicate untreated cells at 6 h and 72 h, respectively. (C) Titration of viral particles released from 72–144 h Dox-treated EREV8 cells. Copy numbers of DNase I-resistant, encapsidated viral DNAs were determined by comparative quantitative PCR of EBV DNA polymerase gene (BALF5) using serial dilutions of Raji DNA as standards. Data are presented as means±SD from six independent PCR assays.

EBV Rta alone is known to disrupt EBV latency in both epithelial and B cells [23], [24]. 293TetER is a recently established 293 cell line that displays doxycycline (Dox) -controlled, conditional expression of EBV Rta [27]. To confirm whether the expression of EBV Rta in 293TetER cells is sufficient to promote EBV lytic replication from the latent stage, rAkata-G418 EBV genome was transferred into 293TetER cells, yielding an EREV8 derivative line [28]. To measure the induction rate of the EBV lytic cycle triggered by EBV Rta, Dox (50 ng/ml)-treated EREV8 cells were analyzed using an immunofluorescence assay and flow cytometry. As expected, the immunofluorescence assay showed a very high and homogenous expression of transgene Flag-EBV Rta (Figure 1A), and flow cytometry showed that ≈76% of the cells were positive. Similarly, considerable expression of immediate-early protein BZLF1 (≈82%) and late glycoprotein protein BALF4/gB (≈50%) were detected in the Dox-treated EREV8 cells (Figure 1A). Next, the expression kinetics of a panel of lytic proteins including BZLF1, BMRF1, BHRF1, and membrane protein gp350/220 in EREV8 cells were compared in parallel using western blot analysis. Although the degree of antibody affinity may vary, the overall kinetics of the different proteins was distinguishable (Figure 1B). The optimal expressions for each protein were in a hierarchical order: namely Flag-Rta (24–48 h), EBV immediate-early protein BZLF1 (48–72 h), early proteins BMRF1 and BHRF1 (72–144 h), and late membrane protein gp350/220 (120–144 h). Finally, the quantity of EBV genome equivalents encapsidated in the viral particles released from Dox-treated EREV8 cells at each time points were determined by comparative quantitative PCR (q-PCR). As shown in Figure 1C, the Dox-treated EREV8 cells continued to produce viral particles in an increasing manner until 144 h. Noticeably, at 96 h after induction, a subpopulation of cells started to round up and seemed to be full of granules. Anoikis-like detachment of cells from the petri dish was observed at later times (detailed below). These observations are reminiscent of proficient permissive replication of bacteriophage in *E. coli*.

**Figure 2:**
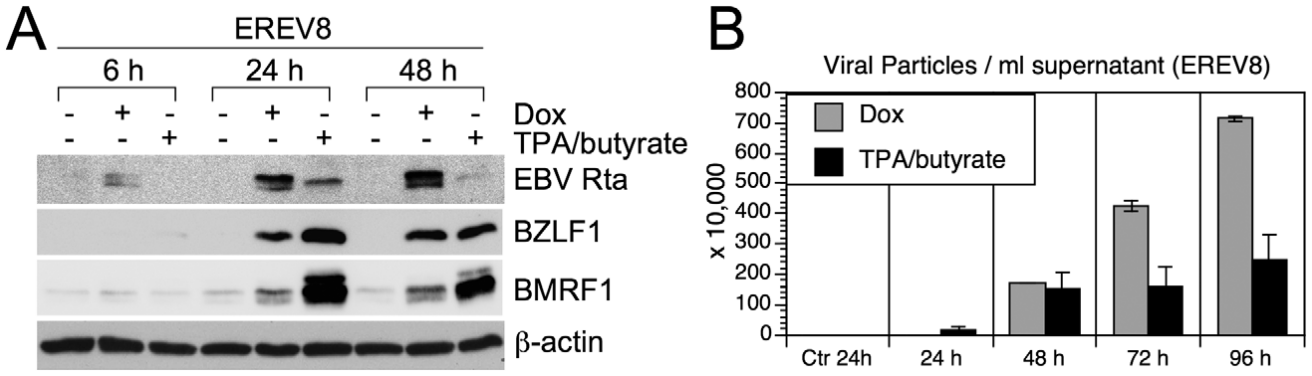
Comparative studies of EBV reactivation induced by Dox (50 ng/ml) vs. conventional chemical method (20 ng/ml TPA plus 3 mM butyrate) in EREV8 cells. (A) Expression kinetics of EBV Rta, BZLF1 at 6, 24, and 48 h in cells treated with indicated inducers. β-actin served as a loading control. (B)Viral particles released from Dox-treated or TPA/butyrate-treated EREV8 cells. Data are presented as means±SD from four independent PCR assays.

To compare the “throughput” of lytic cycle replications initiated by the present system with that by conventional method (20 ng/ml 12-*O*-tetradecanoylphorbol-13-acetate (TPA) plus 3 mM butyrate), EREV8 cells treated with either method were performed simultaneously for a course of 96 h, a time when most of the chemical-treated cells were dead. Of note, in an immunofluorescence assay, while closed to 70% of Dox-treated cells expressed Flag-Rta at 24 h, only 43% of the chemical-treated EREV8 cells responded to TPA/butyrate (i.e. BZLF1 positive), indicating the presence of a considerable refractory subpopulation in the traditional induction system. For each time point, the expressions of EBV immediate-early protein Rta, BZLF1, and early protein BMRF1 were revealed by western blot analysis (Figure 2A) and the yields of encapsidated viral particles were determined by comparative q-PCR (Figure 2B). Interestingly, while Rta was detectable as early as 6 h and increasingly augmented until 48 h in the Dox-treated cells, Rta was only transiently upregulated at 24 h in the TPA/butyrate-treated cells. By contrast, the expressions of BZLF1 and BMRF1 were significantly augmented in the chemical-treated cells than in the Dox-treated group by 48 h. Furthermore, although TPA/butyrate-induced EREV8 cells appeared to shed EBV particles at earlier time (24 h), the overall viral yields in the Dox-treated cell were ≈1.5-higher than that in the chemical-treated cells at 96 h (Figure 2B). In addition, since the Dox-treated cells may continue to shed viral particles at an increasingly manner until 196 h (Figure 1C), it is estimated that from same number of EREV8 cells, the viral particles produced by Dox treatment will be at least 4-fold more than that by chemical induction. Taken together, these results indicated that although TPA/butyrate provided a faster and stronger stimulus for EBV reactivation in the EREV8 cells, yet the induction rate was poorer, the cells were sicker and the viral yield was lower than those triggered by Dox-inducible Rta.

### EBV Rta alone is sufficient to initiate and complete lytic KSHV replication in ERKV cells

**Figure 3:**
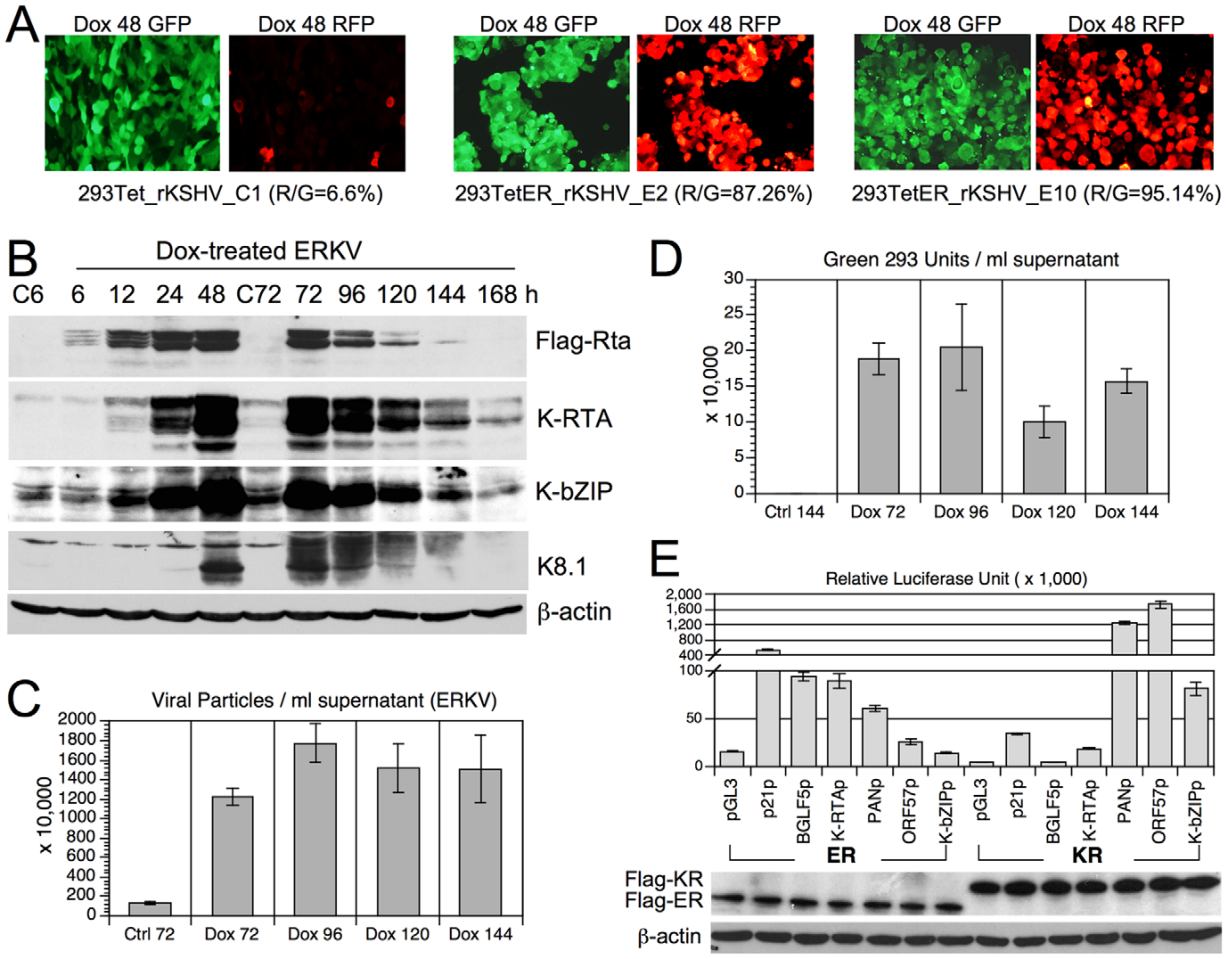
Reactivation of KSHV lytic replication by EBV Rta in ERKV cells. (A) Latent and lytic infections of rKSHV.219, indicated by GFP and RFP, respectively, were inspected in control 293Tet (293Tet_rKSHV_C1) and 293TetER cells (293TetER_rKSHV_E2 and E10) treated with doxycycline (Dox) for 48 h. R/G represents the fraction of RFP-expressing cells in the population determined by using Image J (NIH). Five 293TetER_rKSHV subclones with R/G >70% were pooled and collectively referred to as ERKV. (B) The expression kinetics of KSHV lytic proteins (K-RTA, K-bZIP, K8.1) in control (C6 and C72) and Dox-treated (6–168 h) ERKV cells were examined by western blot analysis. (C) Titration of KSHV particles released from Dox-treated ERKV cells. Copy numbers of DNase I-resistant, encapsidated viral DNAs in each filtrated (0.45 µm) viral supernatants were determined by comparative quantitative PCR of KSHV DNA polymerase gene (ORF9) using serial dilutions of cosmid GB11 DNA as standards. Data are presented as means±SD from six independent PCR assays. (D) Titration of infectious KSHV particles from Dox-treated ERKV cells. Aliquots of filtrated supernatants were used to infect fresh 293 cells. Two days after infection, the numbers of GFP-positive cells, designated as “green 293 units”, in each infection were counted under a fluorescence microscope. Error bars depict standard deviations of three independent counts. Two independent experiments were performed, one set of results is shown. (E) A luciferase reporter gene assay was used to screen the responsiveness of various viral and cellular promoters to EBV Rta (ER) and K-RTA (KR) in 293 cells. The error bars of each column indicate the standard deviation of each set of triplicate wells. The transfection efficiency of each sample was validated by Western blot analysis using M2 Flag monoclonal antibodies.

In the course of establishing EREV8, a control experiment was performed in which rKSHV.219 [29] was used to infect 293TetER cells and served to differentiate the specificity of EBV Rta for its cognate viral genome, as described previously [25]. rKSHV.219 carries a genetic cassette that can be used to distinguish the stages of viral infection in the host cells: latent (green fluorescence) and lytic (red fluorescence) [29]. At first, we expected that Dox-induced EBV Rta would reactivate EBV but not KSHV genomes residing in the 293TetER cells. Somewhat surprisingly, upon the administration of Dox, a number of 293TetER cell clones harboring rKSHV.219 genomes exhibited strong red fluorescence, indicating lytic replication (Figure 3A). By contrast, only a low percentage of Dox-treated control 293Tet cells harboring rKSHV.219 genome exhibited red fluorescence (Figure 3A, 293Tet_rKSHV_C1), suggesting that EBV Rta was the determinant that triggered rKSHV.219 lytic cycle replication. To verify these observations further, five 293_TetER_rKSHV.219 cell clones were expanded, pooled, and collectively designated as ERKV. Stable latent infection of rKSHV.219 in ERKV cells was achieved by puromycin selection using previously described procedures [29]. Next, the expression kinetics of KSHV immediate-early protein K-RTA, early protein K-bZIP, and late protein K8.1 were studied by western blot analysis. Again, the overall expression pattern of these four molecules could be arranged in a cascade manner by their respective peak times: namely Flag-EBV Rta (24–48 h), immediate-early K-RTA and K-bZIP (48–72 h), and late glycoprotein K8.1 (72–96 h) (Figure 3B). Interestingly, expression of the three KSHV lytic proteins was extinguished at 168 h, suggesting no resources were available for virus multiplication. The titers of KSHV particles released into the culture medium at different time points were determined by comparative q-PCR of cell-free, encapsidated KSHV genome equivalents. The results showed that viral particles manufactured in ERKV cells were about 3-fold to that produced by EREV8 cells at 96 h (18 vs. 6 millions/ml), however, the production was plateaued afterwards (Figure 3C), reinforcing cellular resources for KSHV replication were exhausted after 96 h.

To determine the infectivity of these viral particles, an aliquot of the filtrated supernatant was used to infect fresh 293 cells, and the green fluorescence-glowing cells, dubbed as “green 293 units” were determined by fluorescence microscopy (Figure 3D). The highest titer produced at Dox 96 h, 1.8×10^5^ units/ml, is ≈30-fold higher than that induced by the combination of sodium butyrate and K-RTA in the same 293 background described previously (Fig. 8D in [29]), indicating that this new system to induce lytic KSHV replication is very robust. Furthermore, since K-RTA is by far the only known immediate-early protein that is required and sufficient to complete a lytic cycle replication, to confirm whether K-RTA is the only gene activated by EBV Rta in ERKV cells, luciferase reporter gene assays were used to analyze the responsiveness to the cotransfected EBV Rta proteins of a panel of KSHV viral promoters. Two known responders of EBV Rta, namely promoters of EBV BGLF5 and cellular p21, were included as controls. As shown in Figure 3E, the EBV BGLF5 and cellular p21 promoter sequences were responsive to EBV Rta, whereas the three KSHV lytic promoters (PANp, ORF57p, and K-bZIPp) were preferentially responsive to K-RTA, as expected. These results established the respective specificity of EBV Rta and K-RTA for their cognate responsive elements in the present assay. Intriguingly, when the promoter of K-RTA was considered, even though the expression of EBV Rta was much less than that of K-RTA, the K-RTA promoter exhibited a significantly stronger responsiveness to EBV Rta than to K-RTA. Taken together, these results suggest that in Dox-treated ERKV cells, EBV Rta efficiently up-regulates the expression of K-RTA, followed by the activation of numerous KSHV lytic promoters and DNA replication elicited by K-RTA itself. In summary, unexpectedly, we found that EBV Rta alone is also sufficient for initiating and completing the lytic replication of KSHV in 293 cells. Similar to EREV8, ERKV cells consistently became aggregated and disrupted on the fourth day after Dox induction, suggestive of permissive viral replication in these cells.

### Long-term Dox-treated EREV8 and ERKV cells displayed growth arrest followed by cell death

**Figure 4:**
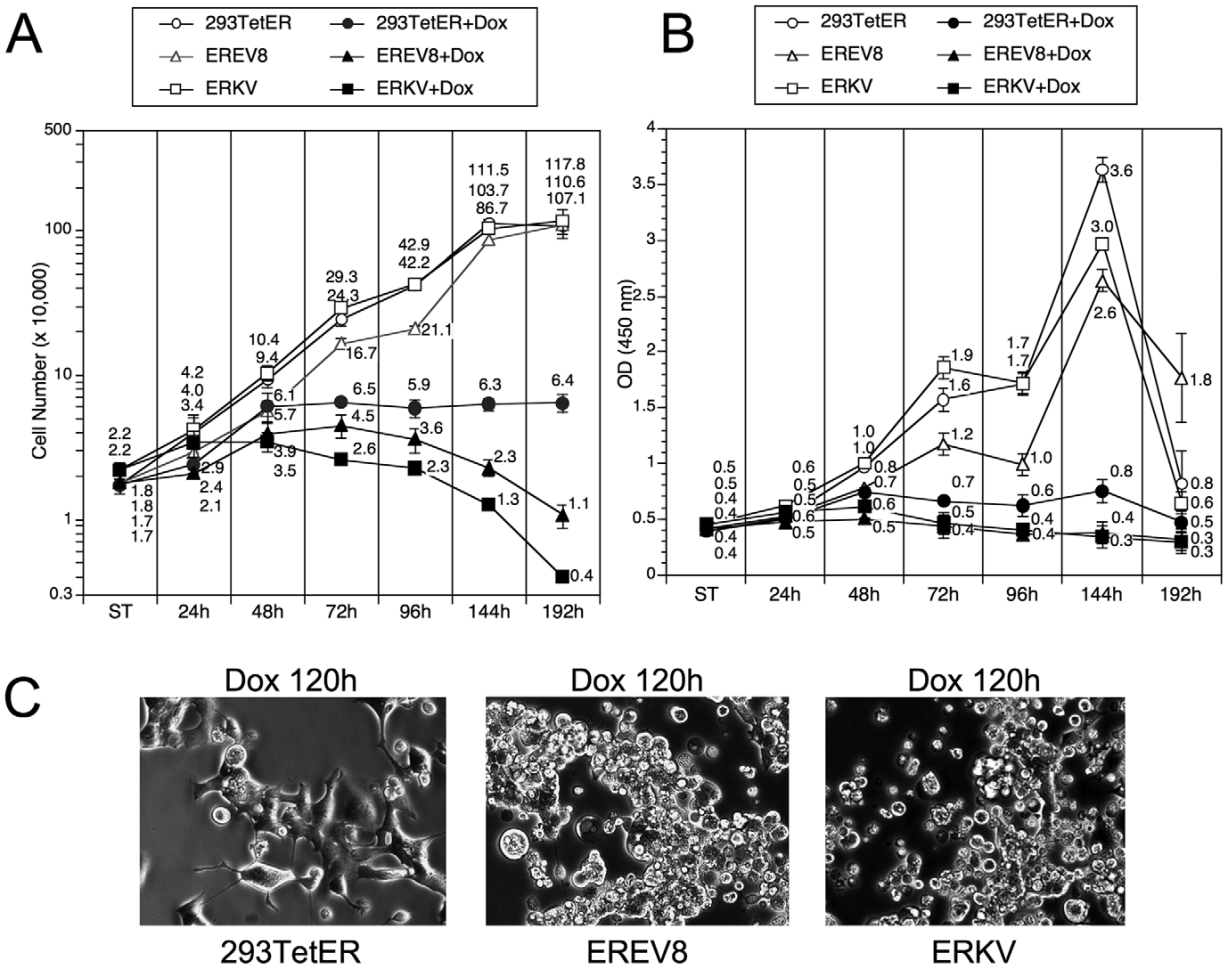
Growth curves and metabolic rate of 293TetER, EREV8, and ERKV cells with or without Dox treatment. (A) Cell growth rates of 293TetER, EREV8, and ERKV were determined by seeding triplicate wells (20,000 cells/well) in a 24-well tissue culture plate for each time point. In parallel, duplicate plates were prepared, and 50 ng/ml Dox was added to each sample 24 h after cell seeding. At the specified times, cells were counted using the trypan blue-exclusion method. Error bars denote standard deviations of triplicate wells. Four independent experiments were performed, one set of representative results is shown. ST, starting numbers. (B) Cell metabolic activity was measured using the WST-1 assay. Cell preparations and Dox treatment were identical to the procedure described in (A). Three independent experiments were performed, and one set of representative results is shown. (C) Disrupted morphologic changes were observed in 120 h Dox-treated EREV8 and ERKV cells, but not in 293TetER cells that exhibited cellular senescence.

We demonstrated previously that EBV Rta can initiate a sustained and irreversible G1 arrest, a hallmark of cellular senescence in both 293 and NPC cells [27]. In the current study, we observed that EBV Rta alone is sufficient to induce and complete the lytic cycle of EBV and KSHV latent genomes in 293TetER cells. To characterize further the molecular phenotypes imposed by EBV Rta-mediated processes, the growth curves and metabolic activities of Dox-treated and -untreated 293TetER, EREV8, and ERKV were followed for eight days. As depicted in Figure 4A, the growth curves for 293TetER, EREV8, and ERKV were similar. A noticeable growth plateau was observed at 144 h, indicating the time when the cells were confluent and the nutrient was depleted from the culture media. In the Dox-treated group, the number of 293TetER, EREV8, and ERKV cells continued to increase until 48 h, after which 293TetER cell count remained stable through the end of the experiment, when the Dox-treated 293TetER cells became senescent, as described previously [27]. By contrast, the number of live Dox-treated EREV8 and ERKV cells declined gradually from 72 to 192 h, suggesting that cell death occurred during this time. In parallel, the metabolic activity of each cell line under each treatment was measured using a WST-1 assay (Figure 4B). In general, the metabolic activities were consistent with the cell number counts. In the untreated group, all three lines exhibited a growth peak at 144 h, followed by an abrupt drop at 192 h, indicating nutrient deprivation and culture confluence. The metabolic activity was maintained in senescent 293TetER cells (a key feature of senescence ([30], [31]), but the metabolic activity in Dox-induced EREV8 and ERKV cells increased in the first 48 h and then declined progressively from 72 to 192 h (Figure 4B). Taken together, these results established that without Dox treatment, 293TetER, EREV8, and ERKV cells exhibit similar growth rates; yet, after Dox induction, EBV Rta led the three cell lines to different fates: 293TetER cells became senescent whereas EREV8 and ERKV cells died eventually. The different cell fates of Dox-treated 293TetER and 293TetER cells containing viral genomes (EREV8 and ERKV) were reflected in distinct cell morphologic changes. Specifically, disruptively rounded-up and anoikis-like cells started to be detectable in 96 h Dox-treated EREV8 and ERKV cells, and were especially prominent from 120 to 192 h (Figure 4C). By contrast, during the same time course, 293TetER cells remained flattened and enlarged without further changes in cell shape (Figure 4C and [27]). These results suggest that the permissive lytic replications of EBV and KSHV in EREV8 and ERKV cells, respectively, may be the main cause of cell death.

### Rta modulated the expressions of G1 arrest genes in 293 and nasopharyngeal carcinoma (NPC-TW01) cells

In addition to 293TetER, we have recently established Tet-on Rta inducible system in NPC cell background, referred to as TW01TetER. As a step to dissect Rta's role in cell cycle, genome-wide transcriptome analysis was conducted in Dox-treated 293TetER and TW01TetER cells (GEO accession # GSE24587). There were 120 genes commonly modulated by Rta in these two inducible cell lines (fold-change ≥1.9). Among these, more than 90% (109 out of 120) were modulated in the same direction in both cell lines. In addition, the inductions of FASN and MERTK were consistent with a previous microarray study (n = 5,000) in which human keratinocytes were infected with adenovirus vector expressing Rta [32], [33]. Thus, some of the transcriptional acts imposed by Rta may be conserved in different cell types. Next, gene ontology analysis [34], [35] was employed to classify these candidates into functionally-related gene sets. As such, the cell cycle-related genes were revealed with p-value≈0.01 in both analyses (Table 1). Interestingly, although some of the genes, e.g. CDK6 in TW01TetER (Table 2), whose expressions were not altered significantly at the mRNA level, we were able to confirm the expressions of five G1 arrest-related genes in both cell types by western blot analysis, including CCND2, CDK6, c-Myc, p21 and 14-3-3σ (detailed below). Therefore, Rta-induced G1 arrest seemed primarily originated from the transcriptional level.

**Table 1:** Partial lists of gene ontology analyses of 120 genes commonly modulated by Rta in 293 cells and NPC-TW01 cells.

Common_120_DAVID [34]	Gene no.	p-Value
regulation of cell proliferation (GO:0042127)	15	1.43E-04
response to organic substance (GO:0010033)	14	2.23E-04
response to mechanical stimulus (GO:0009612)	5	2.99E-04
tissue morphogenesis (GO:0048729)	7	5.93E-04
regulation of apoptosis (GO:0042981)	14	6.35E-04
positive regulation of macromolecule metabolic process (GO:0010604)	14	1.15E-03
response to virus (GO:0009615)	5	3.60E-03
regulation of DNA metabolic process (GO:0051052)	5	4.23E-03
wound healing (GO:0042060)	6	4.89E-03
response to steroid hormone stimulus (GO:0048545)	6	5.00E-03
//		
**cell cycle** (GO:0007049)	11	1.34E-02

Common_120_WebGestalt [35]	Gene no.	p-Value
response to stress (GO:0006950)	24	1.00E-03
organ morphogenesis (GO:0009887)	14	1.70E-03
regulation of cell proliferation (GO:0042127)	14	1.70E-03
response to organic substance (GO:0010033)	13	2.00E-03
response to chemical stimulus (GO:0042221)	18	3.60E-03
epithelium development (GO:0060429)	7	6.00E-03
anatomical structure morphogenesis (GO:0009653)	17	6.10E-03
regulation of DNA metabolic process (GO:0051052)	5	6.10E-03
response to steroid hormone stimulus(GO:0048545)	6	6.50E-03
response to virus (GO:0009615)	5	6.50E-03
//		
**cell cycle** (GO:0007049)	13	1.09E-02

Identified GO terms are sorted by p-Value.

**Table 2:** List of cell cycle related genes modulated by EBV Rta in 293 and NPC-TW01 cells.

Gene Symbol	293TetER	TW01TetER	Gene Description
CCND1	−3.1#	−1.1	cyclin D1
CCND2	−3.1	−3.3	cyclin D2
CDK4	−1.3	−2.2	cyclin-dependent kinase 4
CDK6	−1.8	−1.1	cyclin-dependent kinase 6
CDKN1A	2.2	1.0	cyclin-dependent kinase inhibitor 1A (p21, Cip1)
CHEK2	−1.2	−1.9	CHK2 checkpoint homolog (*S. pombe*)
GNL3	−2.0	−2.1	guanine nucleotide binding protein-like 3 (nucleolar)
H1F0	3.8	2.4	H1 histone family, member 0
HERC5	2.2	1.9	hect domain and RLD 5
HEXIM1	2.3	1.3	hexamethylene bis-acetamide inducible 1
HSPA2	2.8	2.3	heat shock 70kDa protein 2
IFITM1	5.0	1.8	interferon induced transmembrane protein 1
LMLN	1.9	2.2	leishmanolysin-like, metallopeptidase M8 family
MYC	−3.0	−4.5	v-myc myelocytomatosis viral oncogene homolog
NEFH	6.6	1.3	neurofilament, heavy polypeptide
NEFL	−2.3	−3.1	neurofilament, light polypeptide
NOLC1	−1.8	−2.4	nucleolar and coiled-body phosphoprotein 1
PTTG2	1.1	2.0	pituitary tumor-transforming 2
RRS1	−2.6	−2.5	RRS1 ribosome biogenesis regulator
SFN	14.3	1.6	stratifin (14-3-3σ)
TGFB2	−2.1	−1.3	transforming growth factor, beta 2
FASN##	2.2	2.2	fatty acid synthase
MERTK##	8.3	21.5	c-mer proto-oncogene tyrosine kinase

# : Fold-change compared to the control groups. Details for the experimental design and data process procedures are referred to Gene Expression Omnibus (GEO) under accession number GSE24587.

## : Also observed in primary keratinocytes transduced by adenovirus vector expressing Rta [32], [33].

### Comparative analysis of Rta-mediated G1 arrest and viral reactivation

**Figure 5:**
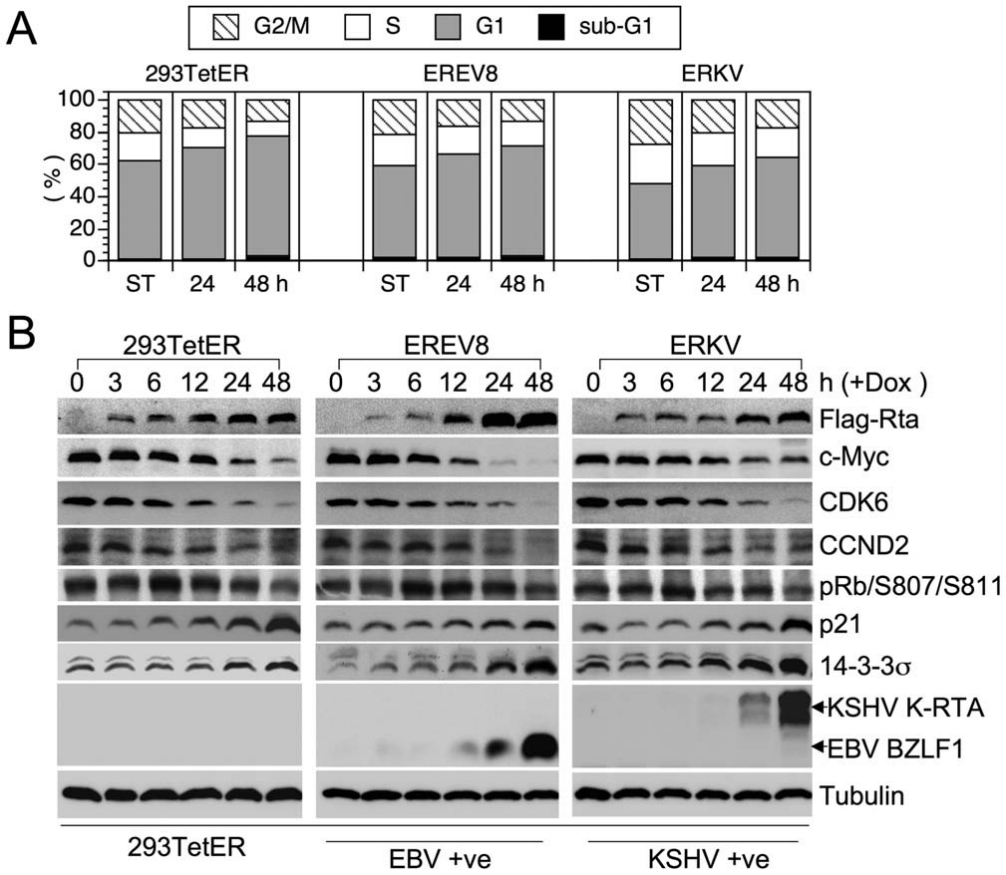
Rta-mediated cell cycle arrest precedes the expressions of viral immediate-early genes. (A) Dox-treated 293TetER, EREV8, and ERKV cells cultured for 24 and 48 h were subjected to flow cytometry analysis to quantify the cellular DNA content. The distributions of cells residing in the G2/M, S, G1, and subG1 stages at each time are shown. The results of three independent experiments were similar, and one representative dataset is shown. (B) Comparative expression kinetics (0–48 h) of cell cycle regulators or viral immediate-early proteins in Dox treated 293TetER, EREV8 and ERKV cells. Down-regulation of cell cycle activators (c-Myc, CDK6, CCND2, phosphorylated pRb) and up-regulation of cell cycle inhibitors (p21, 14-3-3σ) are temporally associated with the expression of Rta in all three cell lines. In comparison, EBV BZLF1 and KSHV K-RTA are not significantly augmented until 48 h, a time that alterations of cell cycle gene are nearly completed. α-tubulin served as a loading control.

Previous results showed that Rta universally modulates the expressions of G1 signature proteins in 293 and NPC cells. These alterations not only support the idea that senescence is preceded by an irreversible G1 arrest [30], [36], but also are reminiscent of a common function of herpesviral immediate-early genes, namely to halt cell cycle progression in G1 [18]. Therefore, we questioned whether G1 arrest was maintained in Dox-induced EREV8 and ERKV cells. To this end, short-term Dox-treated 293TetER, EREV8 and ERKV cells were subjected to flow cytometric analysis. As depicted in Figure 5A, in all three cell lines, G1-populations are increasingly proportional to the Dox induction time, indicating that regardless of viral genomes, Rta-mediated G1 arrest was sustained in both EREV8 and ERKV cells. In order to dissect the time sequence of Rta-mediated host G1 arrest and viral reactivations, we compared the expressions of cell cycle related genes to that of the viral lytic switches. As shown in Figure 5B, in all three cells lines, the decreased expressions of c-Myc, CDK6, CCND2 and increased expressions of p21, 14-3-3σ were temporally associated with the concentration of Dox-inducible Rta between 6 and 48 h. In addition, phosphorylated pRb (S807/S811) was accordingly diminished in all three cell lines, a strong indication of G1 arrest. In marked contrast, an evident induction of viral lytic switch genes, namely BZLF1 in EREV8 and K-RTA in ERKV cells, always lagged behind cellular gene alterations, suggesting that Rta-mediated host G1 arrest preceded the onset of viral reactivation. It is worth noting that the decrement of c-Myc before induction of K-RTA (Figure 5B, ERKV) agrees with a recent report in which elimination of c-Myc led to KSHV reactivation in primary effusion lymphoma cells [37]. Finally, by taking advantage of fluorescence markers residing in the rKSHV.219 genome, we observed that in the Dox 120 h-treated ERKV group, the remaining adherent cells with SA-β-Gal positivity were mostly void of virus lytic replication (not shown), indicating that exhibition of senescence marker and KSHV reactivation were mutually exclusive. Taken together, these results led us to hypothesize that in 293 cells, the Dox-inducible Rta efficiently induces a G1 arrest (6–48 h) that is an ideal environment for EBV or KSHV lytic cycle progression (48–168 h) and is a favorable preceding event for cellular senescence (120 h – ∞).

## Discussion

EBV Rta is a transcriptional activator with high plasticity in viral genome recognition [38], [39]. Results from microarray analysis in different cell backgrounds suggest that Rta also binds to and efficiently modulates the expression of host genome ([32], [33] and Table 2). Here, we investigate the sequential events when Rta encounters host and viral genomes at the same time. First, it is confirmed that Rta efficiently modified the expressions of key cell cycle regulators of which three are related to cellular senescence (c-Myc [16], p21 [30], and 14-3-3σ [40]) (Figure 5B). Second, we observed that Rta-mediated cellular gene alterations preceded the induction of viral immediate-early genes BZLF1 and K-RTA. These phenomena strongly suggested that Rta may utilize a consensus theme to control cell cycles and viral reactivations.

Our results support some previous findings [20], [41], [42], [43], [44], [45] and disagree with some other ones [46], [47], [48]. Rodriguez *et al.* demonstrated that a significant G1 bias was associated with early stages of chemically-induced EBV lytic cycle progression in NPC and B cells [43]. Kudoh *et al.* showed that induction of EBV lytic replication in Tet-On BZLF1 B95-8 cells completely arrested cell cycle progression at G1/S transition and blocked cellular DNA synthesis [20]. When a single gene system is concerned, both EBV BZLF1 and KSHV K-bZIP elicited distinct pathways to arrest host cell cycle in G1 stage in various cellular backgrounds [41], [42], [44], [45]. Thus, our results suggest that the Rta-induced G1 arrest in EREV8 and ERKV cells indeed provided an adequate environment for virus reactivation. By contrast, Zacny *et al.* [48] and Swenson *et al.* [47] observed that Rta interacted with pRb that in turn released E2F1 and activated an S phase in Akata cells, U-2 OS cells and contact-inhibited fibroblasts. Guo *et al.* reported that over-expressions of BZLF1 or Rta in Raji cells resulted in degradation of pRb, accumulation of E2F1 and promotion of S phase entry [46]. It bears to note that in our system the concentrations of E2F1 and pRb were not dramatically modulated by the Dox-inducible Rta ([27] and unpublished). Thus, different cellular context may account for these discrepancies and more experiments are required to resolve this puzzle.

Permissive EBV or KSHV replications have been previously demonstrated in differentiated cells *in vivo* and *in vitro* [4], [5], [7], [8], [49], [50]. In contrast, it is less clear for herpesviruses replicating in senescent cells. So far, papillomavirus E2 [51], human cytomegalovirus IE2 [22] and EBV Rta [27] are the only known viral products involved in cellular senescence. E2 was previously shown to induce cellular senescence in HPV infected HeLa cells by restoring the functions of p53 and pRB [52], [53]. Whether IE2 or Rta-induced cellular senescence contributes to viral pathogenesis *in vivo* is worthy of further investigation. Of note, since both BZLF1 and Rta possess G1 arrest function, a synergistic effect of BZLF1 and Rta in cell cycle arrest is expected. Furthermore, Kalla *et al.* recently demonstrated that BZLF1 and Rta were expressed as immediate-early genes following primary EBV infection of B lymphocytes [54]. However, these early-expressed BZLF1 and Rta failed to initiate the EBV lytic cycle owing to the intruding viral genome was in an un-methylated status [54]. Therefore, we hypothesize that in such a transient-lytic phase where only the host genome is accessible, Rta (and BZLF1) may exert to trigger a cell senescence process.

Among genes modulated by Rta depicted in Figure 5B, the sharply decreased expression of c-Myc by EBV Rta has two implications. First, one oncogenic role of c-Myc was suggested to be a repressor of cellular senescence [16], [55]. In our previous report, we demonstrated that EBV Rta efficiently induces cellular senescence in 293, NPC-TW01 and HONE-1 cells [27]. Here we further confirm that in these senescent cells the decrement of c-Myc was one of the earliest events modulated by EBV Rta. Thus, decreased expression of c-Myc via Rta seems to participate in Rta-induced cellular senescence. Second, c-Myc is a negative regulator of KSHV lytic cycle replication [37], [56]. RNAi-mediated knockdown of c-Myc resulted in disruption of KSHV latency and increment in mRNA and protein levels of K-RTA [37]. Consistent with these results, we observed that the reactivation of KSHV latent genome was preceded by a gradual decrement of c-Myc in the ERKV cells (Figure 5B). In addition, in a luciferase-reporting assay, the promoter sequences of K-RTA, but not those of K-bZIP, PAN, and ORF57 were preferentially activated by ectopic expression of Rta (Figure 3E). Thus, we hypothesize that either a direct act from Rta alone, or via down-regulated c-Myc, or both, are attributable to Rta-induced K-RTA synthesis in Dox-treated ERKV cells.

EBV Rta is not the only variant that cross-reactivates KSHV; other viral factors including the HCMV *UL112-113* locus [57] and HIV-1 *tat* protein [58] have also been ascribed to possess such functionality, suggesting that other viral infections may also participate in KSHV pathogenesis. Further, although permissive EBV or KSHV lytic replication were detectable *in vivo*, but a homogenous and thorough lysis of host cell by viral lytic replication is still lacking *in vitro*. Here, we have produced a model that provides a nearly permissive replication system for both EBV and KSHV that is controlled directly by EBV Rta. This system offers two advantages over the conventional approaches. First, the stimulus, 50 ng/ml Dox, is a very dilute, physiologically neutral compound. Compared with the conventional sodium butyrate or phorbol ester, Dox elicits far fewer, possibly no, undesirable effects on the treated cells. Second, the treatment produces homogenous results. Routinely, Flag-tagged EBV Rta and BZLF1 were detected in close to 80% of the 48 h Dox-treated EREV8 cells when assessed by an immunofluorescence assay. Similarly, more than 80% of the treated ERKV cells produced red fluorescence 48 h after induction. Our newly established EREV and ERKV cells thus provide a feasible system for elucidating host factors and viral determinants that contribute to regulate the EBV and KSHV reactivations.

## Materials and Methods

### Cell culture

293TetER is a doxycycline inducible, EBV Rta conditional expression cell lines created by Virapower system™ (Invitrogen, Carlsbad, CA) [27]. Same procedures were carried out to establish TW01TetER in which inducible Rta was expressed in a nasopharyngeal carcinoma cell line, NPC-TW01 [59]. EREV8 is an EBV positive 293TetER derivative line generated by using cell-to-cell infection method [28]. ERKV is a KSHV positive 293TetER derivative line that was stably infected with rKSHV.219 [29]. Specifically, 293TetER cells incubated with rKSHV.219 viral sup for 48 h were selected with 660 µg/ml puromycin for three weeks to obtain green fluorescent clones. Twelve such cell colonies were isolated, expanded, and determined for inducibility of KSHV lytic replication (red fluorescence) by 50 ng/ml doxycycline treatment. Five clones with high inducibility (70–90%) were pooled and used to compose the first generation of ERKV. 293TetER, EREV8 and ERKV cells were maintained in DMEM containing 10% Tet System Approved FBS (Clontech Laboratories, Mountain View, CA), 5 µg/ml blasticidin-S-HCl (Invitrogen) and 200 µg/ml zeocin (Invitrogen). To maintain the latently infected viral genomes, EREV8 and ERKV cultures were further supplemented with 400 µg/ml G418 and 660 µg/ml puromycin, respectively.

### Plasmids

pLenti4-Flag-CPO is a modified expression plasmid derived from pLenti4/TO/V5-DEST (Invitrogen). In brief, the original attR1 site to V5 epitope region (nt 2405–4203) in pLenti4/TO/V5-DEST was replaced with an in-frame DNA fragment encoding Kozak sequence, ATG, FLAG tag and a rare cutter CPO I site (5′CGGTCCG). Accordingly, the cDNAs of EBV Rta (M-ABA strain) and KSHV RTA (Genebank: U71367.1) were PCR-amplified with CPO I sites flanking at both ends, and subcloned into pLenti4-Flag-CPO. The resulting plasmids, namely pLenti4-Flag-ER and pLenti4-Flag-KR, were propagated in DH5α and used in further studies. The upstream sequences of p21 (2.4 kb), EBV BGLF5 (nt 108641 to 110053 of NC_007605), K-RTA (nt 70240 to 71597 of U75698), PAN (nt 28159 to 28660), ORF57 (nt 81556 to 82005), and K-bZIP (74619 to 74849), were cloned in front of luciferase gene located in pGL3-Basic (Promega, Madison, WI), yielding pGL3-Basic-p21p, -BGLF5p, K-RTAp, -PANp, -ORF57p and -K-bZIPp, respectively.

### Transfection and luciferase reporter assay

Transfection was performed in 24-well plates. The next day when the cultured 293 cells were 90% confluent, appropriate amount of indicated plasmids were transfected into cells by using Lipofectamine™ 2000 (Invitrogen) according to the manufacturer's instructions. Twenty-four hr after transfection, cells were harvested for luciferase activity assay by using Dual-Glo lucifearse assay kit (Promega). In addition, an aliquot of cell lysates was subjected to western blot analysis for the normalization of each transfection efficiency.

### Titration of EBV and KSHV viral particles

Filtrated (0.45 µm) viral supernatant (160 µl) was incubated with 2 U DNase I (Invitrogen) at 37°C for 30 min followed by extraction of encapsidated EBV DNA using QIAamp MinElute virus spin kit (QIAGEN). Each comparative quantitative PCR reaction was composed of 4 µl diluted viral DNA, 5 µl Power SYBR Green Master Mix (Applied Biosystems, Foster City, CA), and 1 µl primer mix (2 µM). The primers used in the present study were as follows: detection of EBV genome, BALF5-forward (5′-CGGAGTTGTTATCAAAGAGGC-3′) and BALF5-reverse (5′-CGAGAAAGACGGAGATGGC-3′); detection of KSHV genome, ORF9-forward (5′-CCAACATCATCCAATGCCTC-3′) and ORF9-reverse (5′-GGGAAAAGTCACGGGAATG-3′). Known copy numbers of serially diluted EBV genome from Raji cellular DNA (50 copies/cell) were used as standards in titrating EBV viral particles. Known copy numbers of serially diluted cosmid GB11 DNA encompassing KSHV genome nt 1–35,022 (U75698) were used as standards in titrating KSHV viral particles. The reaction was conducted and detected by StepOnePlus™ Real-Time PCR system (Applied Biosystems).

### Infectivity assay of KSHV particles

We followed the EBV infection procedure described by Hutt-Fletcher and colleagues with minor modification [60]. Specifically, 293 cells were seeded onto 12-well plates at 1.2×10^5^ cells/well that produced ≅30% confluent monolayer 24 h later. Two hundred-µl undiluted, filtrated viral supernatant was gently applied onto the surface of cells. After 2 h of incubation on cells, 1.5 ml growth medium was added and the cells were reincubated for 48 h. To score the infectious units in each well, the culture supernatant was removed, cells were trypsinized and subjected to visual inspection for GFP expression under a fluorescence microscope (OLYMPUS BX51, Olympus UK Ltd, Essex SS2 5QH, UK).

### Western blot analysis

Cell lysates extracted by RIPA buffer were subjected to SDS-PAGE separation and transferred onto nitrocellulose membranes. The membranes were blocked for 1 h in 1× TBST containing 5% non-fat milk and then incubated with the indicated primary antibody overnight at 4°C. The blots were washed three times with 1× TBST for 5 min each. The blots were incubated with peroxidase-conjugated secondary antibody in blocking buffer for 1 h at room temperature. Blots were washed three times with 1× TBST for 5 min each and developed by SuperSignal West Pico chemiluminescent substrate kit (Pierce).

### Cell proliferation assay

Cell proliferation was determined by using a WST-1 kit (Roche, Indianapolis, IN) or by trypan blue exclusion method as described previously [27].

### Immunofluorescence assay

Cells were resuspended in PBS and dropped onto multiple-well diagnostic microscope slides and fixed in methanol/acetone (1∶1) at −20°C for 20 min. Cells were permeabilized with 0.1% Triton-X 100 at room temperature for 20 min. The slide was incubated with indicated primary antibody at room temperature for 1 h, washed three times in PBS for 5 min each, and incubated with FITC-conjugated secondary antibody at room temperature for 1 h. After PBS wash, the slide was incubated with Hoechst 33258 at room temperature for 20 min, washed with PBS, mounted in VECTASHIELD ™medium and inspected by fluorescence microscopy. To quantitate the percentage of positively immuno-reactive cells in the immunofluorescence assay, an aliquot of cells were analyzed in parallel by flow cytometric analysis.

### Flow cytometric analysis

Cells were harvested by centrifugation, washed with phosphate-buffered saline (PBS), fixed in ice-cold 75% ethanol and stored at −20°C until all samples from different time points were collected. Of note, to quench the green and red fluorescence in ERKV cells, the fixation reagent was replaced with 95% methanol. Prior to flow cytometer analysis, the fixed cells were repelleted by centrifugation, permeabilized in PBS containing 0.1% Triton X-100 at room temperature for 30 min, and resuspended in PBS containing 50 µg/ml propidium iodide and 50 µg/ml RNaseA. After the cells were incubated in dark for 30 min, cell cycle profile analysis was carried out on 5,000 cells with a fluorescence activated cell sorter (FACSCalibur, Becton Dickinson, Franklin Lakes, NJ). The results were analyzed by using WinMDI v2.8 software.

### Antibodies

Mouse monoclonal antibodies of EBV proteins were: Rta (467), BZLF1 (4F10), BMRF1 (88A9), BALF4/gB (L2), BHRF1 (3E8), and gp350/220 (72A1). Anti-KSHV RTA was provided by Dr. Keiji Ueda (Osaka University Medical School, Japan). Anti-KSHV K-bZIP was provided by Dr. Mengtao Li (University of Kentucky College of Dentistry, USA). All other antibodies were commercially available: KSHV K8.1 (ABI, Columbia, MD); CDK6, pRb/S807/S811 and p21 (Cell Signaling Technology, Danvers, MA); c-Myc (Santa Cruz Biotechnology, Santa Cruz, CA); 14-3-3σ (GeneTex, Irvine, CA); CCND2 (BD Pharmingen, Franklin Lakes, NJ); α-tubulin (Millipore, Billerica, MA); β-actin and M2-FLAG (Sigma-Aldrich, St. Louis, MO).
